# Anti-Inflammatory and Analgesic Evaluation of a Phytochemical Intercalated into Layered Double Hydroxide

**DOI:** 10.3390/pharmaceutics14050934

**Published:** 2022-04-25

**Authors:** Viviane A. Guilherme, Vanessa R. R. Cunha, Eneida de Paula, Daniele R. de Araujo, Vera R. L. Constantino

**Affiliations:** 1 Departamento de Bioquímica e Biologia Tecidual, Instituto de Biologia, Universidade Estadual de Campinas—UNICAMP, Campinas 13083-862, SP, Brazil; viguilherme@gmail.com (V.A.G.); depaula@unicamp.br (E.d.P.); 2 Faculdade de Farmácia, Universidade Adventista de São Paulo—UNASP, Engenheiro Coelho 13448-900, SP, Brazil; 3 Departamento de Química Fundamental, Instituto de Química, Universidade de São Paulo—USP, Av. Prof. Lineu Prestes 748, São Paulo 05508-000, SP, Brazil; vanessa.roberta@ifmt.edu.br; 4 Instituto Federal de Educação, Ciência e Tecnologia de Mato Grosso—Campus Juína—IFT-MT, Juína 78320-000, MT, Brazil; 5 Centro de Ciências Naturais e Humanas, Universidade Federal do ABC—UFABC, Santo André 09210-170, SP, Brazil

**Keywords:** *p*-hydroxycinnamic acid, 3-(4-hydroxyphenyl)-2-propenoic acid, coumaric acid, hydrotalcite-like material, intercalation compounds, anti-inflammatory, analgesic, drug delivery systems

## Abstract

Coumaric acid (CouH), an antioxidant molecule assimilated by food consumption, was intercalated into layered double hydroxide (LDH) nanocarrier, having zinc and aluminium ions in the layers (LDH-Cou), to evaluate its pharmacological activity through in vitro and in vivo assays in mice. Therefore, the following tests were performed: coumarate delivery in saline solution, fibroblasts’ cell viability using neutral red, peritonitis induced by carrageenan, formalin test, acetic-acid-induced writhing, and tail-flick assay, for the non-intercalated CouH and the intercalated LDH-Cou system. Furthermore, different pharmacological pathways were also investigated to evaluate their possible anti-inflammatory and antinociceptive mechanisms of action, in comparison to traditionally used agents (morphine, naloxone, caffeine, and indomethacin). The LDH-Cou drug delivery system showed more pronounced anti-inflammatory effect than CouH but not more than that evoked by the classic non-steroidal anti-inflammatory drug (NSAID) indomethacin. For the analgesic effect, according to the tail-flick test, the treatment with LDH-Cou expressively increased the analgesia duration (*p* < 0.001) by approximately 1.7–1.8 times compared to CouH or indomethacin. Thus, the results pointed out that the LDH-Cou system induced in vivo analgesic and anti-inflammatory activities and possibly uses similar mechanisms to that observed for classic NSAIDs, such as indomethacin.

## 1. Introduction

Inflammation is a fundamental physiological phenomenon to the organism, started in response to a variety of factors such as mechanic trauma, molecular oxygen deficiency, diet immunological alterations, genetic factors, chemical agents (xenobiotics), physical inactivity, microorganisms, extreme temperature value, and ultraviolet radiation exposure [1,2]. However, the persistent physiological response can be associated with the development of pathologies such as autoimmune diseases (as rheumatoid arthritis and osteoarthritis), arteriosclerosis, degenerative diseases, or even cancer [3].

Frequently, the conventional pharmacological therapies used to decrease the inflammation symptoms are glucocorticoids and nonsteroidal anti-inflammatory drugs (NSAIDs) [4,5], whose molecular mechanisms of action involve the inhibition of the cyclooxygenase route, also a common target of a great number of bioactive molecules from plants [6]. The inflammatory process is characterized by heat, pain, flushing, and swelling, which originate from the effects of mediators (interleukins, prostaglandins, chemokines) and inflammatory cells (macrophages, leukocytes) directly on the blood vessels [1].

The inflammatory process is very unpleasant to the patient because it comes up with pain, characterized as an emotional event of uncomfortable feeling, associated or not associated with a tissue lesion [7]. In severe cases, the pain achieves an extreme level and interferes with the patient’s routine. Some terminologies are used to name the different phases of the painful process. The physiological response to an aggressive stimulus is denominated nociception because it encompasses neurotransmitters (nociceptors) and mediators of inflammation, which can be directly evaluated [8]. Pain is, therefore, the sum of external response (painful behaviour and suffering), and the nociception is a pathophysiological response of the organism to an aggressive stimulus. The detection of a painful stimulus is performed by nociceptors present in the superficial tissues of skin, mucosa, and viscera, which have a pain peripheral response when activated or sensitized [7].

The peripheral modulation of the nociceptive response is initiated by the inflammatory process generated by a lesion. The inflammatory mediators released are responsible for the hyperalgesia (decrease of the threshold action of the nociceptors): the prostaglandins PGE2, PGF2, and PGI2 that potentialize the bradykinin effect, and serotonin 5-HT that increases the sensitivity of the nociceptive receptors. The inflammation is also related to other mechanisms stimulated by silent nociceptors that are not sensitive to thermal and mechanical intense stimuli and the increase of the numbers of opioids receptors. Opioid receptors display their physiological effects by the stimulus of morphine, a classic analgesic opioid and clinical reference of that class of drugs. Morphine stimulates the opioids receptors that are antagonized by naloxone [5,7].

The analgesia triggered by the opioids has an immediate response and generally has a more prolonged effect than that induced by the NSAIDs. However, this class of drugs has undesirable effects such as sedation, euphoria, pupillary constriction, reduced gastrointestinal motility, respiratory depression, and chemical dependence [5,7].

Another system related to the analgesia involves the adenosine (A1 and A2) receptors. The A1 receptor stimuli incite antinociceptive effects. Moreover, molecules that act on this receptor, aside from adenosine, trigger an analgesic effect. However, when the stimulation is on the A2 receptors, a pro-nociceptive response is initiated, mediated by cAMP (cyclic adenosine monophosphate) independently of the inflammation mediator [5,9]. The investigation of the A2 route is performed by using A1 receptor antagonists such as caffeine [9,10].

The studies of the anti-inflammatory effects of molecules have also focussed on plant-based bioactive species considering their recognized beneficial health effects. Among the categories of phytochemicals, phenolic acids such as hydroxycinnamic acids and derivatives have been highlighted because of their important biological properties [11,12]. The *p*-hydroxycinnamic acid, usually known as *p*-coumaric acid (CouH), is a secondary metabolite present in vegetables (for instance, beans and potatoes), cereals (such as maize), and fruits (e.g., apple, pear, and orange) [13]. CouH’s main uses, e.g., as a food preservative, a component of food packaging materials, and an electrochemical sensor, are related to its antioxidant property [12,14]. As a biological antioxidant, CouH is a potential molecule to be evaluated in therapies related to oxidative-stress-induced diseases, playing a role as a scavenger of reactive oxygen and nitrogen species in the organism. This phytochemical also shows important properties such as anticancer [15], antibacterial [16,17], antidiabetic and antihyperlipidemic [18], in the treatment of Parkinson’s disease [19], as immunomodulatory and anti-inflammatory [20,21], anti-leishmanial [22], and antiplatelet [23].

The therapeutical benefits of the traditional administrated drugs or phytochemicals are sometimes limited by their physicochemical properties (solubility), chemical stability, poor bioavailability, toxicological effects, and even physiological barriers of the body. The development of drug delivery systems (DDSs) can improve the physicochemical properties of a bioactive molecule, including its therapeutical effects; decrease systemic side effects; and protect it from degradation promoted by light, heat, or biomolecules such as enzymes, in the organism [24,25]. Among the inorganic DDSs, the layered double hydroxide (LDH) materials have been getting distinctive attention because of their high loading capacity. They have the ability to accommodate several classes of drugs between the layers (flexible porous), be administered by diversified routes, be a pH responsive carrier, be obtained under synthetic sustainable conditions, and other properties that enhance the therapeutical effectiveness of the intercalated bioactive [26].

**Figure 1:**
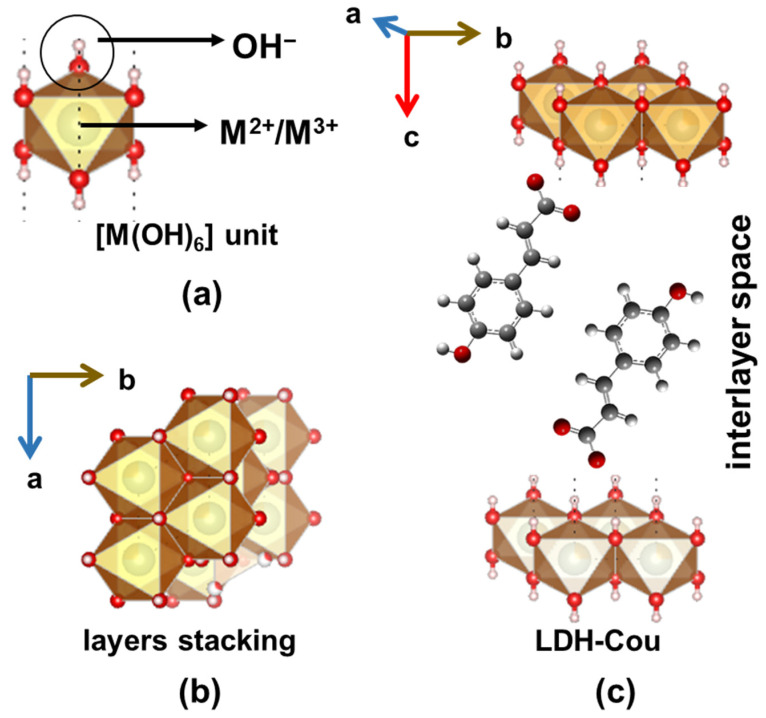
Schematic representation of (**a**) basic octahedral unit in the LDH layers; (**b**) [M(OH)_6_] units are joined by the edges forming layers in a stacked fashion, and (**c**) structure of LDH-Cou (Zn_2_Al-Cou) material. Fractions of LDH structure (ICSD number 91155) were obtained using the software Vesta version 3.

LDH materials have a two-dimensional organized structure represented by the general formula [M^2+^_1−x_M^3+^_x_(OH)_2_]^x+^(A_x/m_)^x−^·nH_2_O, whereas M^2+^ is a bivalent cation, M^3+^ is a trivalent cation, and A^m−^ is the intercalated anion with *m*− charge. The metal cations are in the centre of an octahedral site and are coordinated to six hydroxide anions, as shown in Figure 1a. The [M(OH)_6_] units are joined by the edges, through µ_3_-OH bonds, forming layers that are arranged face-to-face (Figure 1b). The layers are positively charged and the material’s electroneutrality is maintained by negative species in the interlayer region, together with water molecules [27]. The formula is usually abbreviated as M^2+^_R_M^3+^-A, where R is the M^2+^/M^3+^ molar ratio. LDH with the Mg_3_Al-CO_3_^2^ composition is commercialized as antacid, under the general name of hydrotalcite or brand names such as Talcid™ or Gastrum Plux™, for instance [28].

At the end of the 1990s and the beginning of the 2000s, studies reporting the intercalation of anionic drugs into LDH structures were published envisaging the application of this class of inorganic materials as DDSs and, later, theragnostic platforms [26,29]. Several works regarding the intercalation of NSAIDs such as ibuprofen and naproxen into the LDH materials have reported distinct layer compositions and different preparation methods and characterization techniques. On the other hand, biological assays with the intercalated anti-inflammatory drugs are still scarce [30,31,32,33]. We have prepared and performed a deep structural characterization of M_2_Al-Cou (M = Mg^2+^ or Zn^2+^) materials with high loading capacity (about 32–35 wt.%) [34]. The XRD profile refinement of the highly crystalline Zn_2_Al-Cou material and the Le Bail analysis indicated that coumarate ions are arranged as an interpenetrated bilayer, in which negative carboxylate groups are close to the positive layers, as shown in Figure 1c.

Previous in vivo biocompatibility tests carried out with M_2_M′-Cl (M = Mg^2+^ or Zn^2+^; M′ = Al^3+^ and Fe^3+^) samples by tablet implantation in the rat abdominal wall [35,36] were also accomplished with M_2_Al-Cou (M = Mg^2+^ or Zn^2+^) materials. After 28 days of M_2_Al-Cou tablet implantation (tissue remodelling phase), histopathological data showed no antigenic reaction signals or fibrotic encapsulation. The biointegration of the materials with the tissues and the deposition of mainly collagen type-III around the Mg_2_Al-Cou tablet and collagen type-I around Zn_2_Al-Cou were observed. These results motivated advancement in the studies about the therapeutic activity of DDSs based on LDH-Cou. The encapsulation of phenolic phytochemicals can be advantageous because of their low absorption rate, low bioavailability, and reduced stability in the organism [37].

In this work, the performance as a drug delivery system, as well as the anti-inflammatory and antinociceptive properties of LDH-Cou were evaluated in vitro (release assay using Franz diffusion cell; fibroblast cell viability tests) and in vivo assays (peritonitis, formalin, number of writhings, and tail-flick tests). Additionally, different pharmacological pathways were also investigated to evaluate the possible anti-inflammatory and antinociceptive mechanism of CouH and LDH-Cou action, considering traditionally used drugs (morphine, naloxone, caffeine, and the commercial NSAID, indomethacin). Although the anti-inflammatory activity of commercial NSAID is higher than that of CouH or DDS system, the analgesic effect of LDH-Cou obtained by tail-flick tests is very impressive (even the non-intercalated coumarate shows enhanced effect compared to indomethacin).

## 2. Material and Methods

### 2.1. Reagents

All reagents used in this work are listed in the Supplementary Material.

### 2.2. Preparation of LDH-Cou Material

The synthetic procedure to obtain an LDH-Cou sample as well as its detailed characterization (structural, spectroscopic, and thermal) was previously described [29]. According to the chemical elementary analysis and the water content measured by thermal analysis, the chemical formula proposed for the Zn_2_Al-Cou sample is [Zn_2.3_Al(OH)_6.6_](C_9_H_7_O_3_)·1.9H_2_O. The amount of coumarate anion is 34.8 wt.%. In the cytotoxicity tests, a Zn_2_Al-Cl material (abbreviated LDH-Cl), prepared and characterized according to ref. [35], was used for comparison purposes.

### 2.3. In Vitro Assays

#### 2.3.1. Release Kinetics Experiments

The release assay was performed in a Franz diffusion cell with a membrane of cellulose acetate. In the donor vial, 0.5 mL of CouH solution or LDH-Cou suspension was added. Both samples were prepared at equimolar (0.610 mmol L^−1^) coumaric acid concentration. In the receptor compartment, a 30% propylene glycol solution in phosphate buffer at pH 7.5 was added to simulate the intestine fluid. At established time intervals, aliquots of the receptor solution were taken and analysed by UV–VIS spectrophotometry to determine the amount of phytochemical released. The data about the spectrophotometric determination of *p*-coumaric acid concentration in 0.9% of aqueous sodium chloride solution are given in the Supplementary Material (Figures S1 and S2).

The Higuchi model was applied to the kinetic curves of the phytochemical release, which assumes a Fickian diffusion as the rate limiting step [38]. The Higuchi model considers that the fraction of drug released at a defined time (*Q_t_*) is linear in function of the square root of time (*t*). The angular coefficient is the release rate constant (*k_H_*), as shown in Equation (1):

(1) $$Q_{t}=k_{H}t^{\frac{1}{2}}$$

The data regarding the zeta potential and mean particle size measurements are also in the Supplementary Material (Figure S3).

#### 2.3.2. Neutral Red Cytotoxicity Assay

The cell viability was assessed in fibroblasts (NIH 3T3 cell line) by neutral red (NR) uptake test, according to ISO 10993-5: 2009 normative. Cell viability was assessed in murine NIH 3T3 fibroblasts, at the 35th passage. NR is a soluble organic dye molecule that presents the ability to enter in the cells and to be accumulated in the lysosomes [39]. NR is barely retained into the cell when the lysosomal membrane is damaged. Therefore, the amount of dye extracted (quantified by spectrophotometric method) is related to the number of viable cells. For cytotoxicity assays, NIH 3T3 fibroblasts were seeded in 96-well plates (2 × 10^4^ cells/well) in DMEM medium supplemented with 10% foetal bovine serum and 5% CO_2_ at 37 °C and humified atmosphere. After 48 h, the medium was replaced by a solution of coumaric acid or LDH-Cou (or LDH-Cl) suspension in the following concentrations: 0.2, 0.5, 1, 2.5, 5, 7.5, and 10 mmol L^−1^, which correspond to 0.032, 0.08, 0.16, 0.4, 0.8, 1.2, and 1.6 mg mL^−1^ of the organic compound Cou. Hence, Cou-H and LDH-Cou samples were prepared to have the same coumaric acid concentrations while for LDH-Cl assays, used as control, samples contained the equimolar amounts of LDH carrier as in the LDH-Cou samples. After 24 h, the solutions were removed, and the cells were treated with 100 μL of a 50 μg mL^−1^ solution of NR. After 3 h, the NR was removed, and the cells were washed with a solution of phosphate-buffered saline (PBS) to the final addition of 100 μL of an aqueous solution of 50% ethanol and 1% acetic acid. The plate was stirred for 20 min, and absorbance of NR was determined at 540 nm by using Bio-Tek^®^ ELx800 spectrophotometer (Agillent Technologies, Santa Clara, CA, USA).

### 2.4. In Vivo Assays

#### 2.4.1. Anti-Inflammatory and Analgesic Evaluation

Male Swiss adult mice, weighing 30 to 35 g were used for these studies. The animals were provided by the Animal Care Centre of the State University of Campinas (Centro de Bioterismo, CEMIB-State University of Campinas, UNICAMP, Campinas, São Paulo, Brazil), and maintained in light/dark cycles of 12 h, with water and feed ad libitum, in a monitored temperature of 22 ± 3 °C, collectively placed (*n* = 7/experimental group, 5 animals per cage) and acclimatized to the experimental location for at least 7 days. Protocols were approved by the Ethics Committee on Animal Experimentation (Comissão de Ética na Experimentação Animal, CEEA-UNICAMP) accordingly to the codes 2134-1, 2135-1, and 2136-1 (13 May 2010). For pharmacological assays, the experimental groups were organized as follows: (i) Groups 1 to 3 received CouH and Groups 4 to 6 were treated with LDH-Cou at 10, 20, and 30 mg/kg, respectively; (ii) Group 7 was treated with indomethacin (Ind) at 50 mg/kg, Group 8 received 0.9% saline, Group 9 was treated with 7.5 mg/kg morphine (Morph), Group 10 was treated with 5 mg/kg naloxone (Nalox), and Group 11 received 10 mg/kg caffeine (Caffe). Ind concentration was the same as the commercial formulation (50 mg/kg) while the Nalox and Morph concentrations were chosen based on previous work [40].

##### Anti-Inflammatory Evaluation: Peritonitis Induced by Carrageenan

The analysis of the peritoneal exudate allows an inflammatory cell count after exposure to a harmful stimulus. Therefore, it is possible to determine the activity of each compound over the migration of these inflammatory cells [41]. After 1 h of drug treatment, 0.1 mL/10 g of 1% carrageenan solution in 0.9% sterile NaCl was injected intraperitoneally. After 4 h, the animals were sacrificed, and the peritoneum cavity was filled with 2 mL of 5 mmol L^−1^ PBS solution, pH = 7.4. All the exudate was collected and transferred to a microtube. An aliquot of 20 µL was added to 0.4 mL of Turck’s solution with vigorous agitation to avoid cell agglomeration. The cell counting using an Optical Microscope (Nikon Eclipse TS100, Tokyo, Japan) with 10× magnitude) was performed by the addition of 10 µL of the samples in a Neubauer Chamber.

##### Anti-Inflammatory and Analgesic Evaluation (Antinociceptive Assay): Formalin Assay

The formalin assay allows the verification of the anti-inflammatory and analgesic effects of different substances over a nociception activation [40]. The pain behaviour is determined by the time of inflamed paw lick, as: neurogenic pain (0 to 5 min), interphase (5 to 15 min), and inflammatory pain (15 to 30 min) by the number of licks [42]. Here, the animals were treated with the drugs described above (groups 1–10, Section 2.4.1) and with 7.5 mg/kg of Morph to verify the opioid system’s participation in the pharmacological effects of the tested compounds 1 h before the subplantar injection of 20 mL of 1% formalin solution in the right paw. Afterwards, the number of licks were computed as neurogenic or anti-inflammatory pain, whereas the interphase animals did not lick their paws. In the second test, the administration—by intraperitoneal injection—of 5 mg/kg of Nalox, 30 min prior to the subplantar injection of formalin and 30 min after the administration of the compounds to be analysed was evaluated. A third test was conducted by the administration of 10 mg/kg of Caffe, using the same procedure described above.

The association with Nalox was essential to evaluate the possible coumaric acid action in the opioid system, since opioids are described as compounds that present an effect similar to that of Morph and that are blocked by its antagonist, Nalox [43,44]. The administration of Caffe was important to verify the signalling mechanism with cAMP participation [10].

##### Analgesic and Antinociception Evaluation: Acetic-Acid-Induced Writhing

This assay allowed the evaluation of the analgesic and antinociception effects of CouH and LDH-Cou, by comparison of the total number of writhings among the different treatments. Initially, the animals were treated with the above-mentioned groups (Section 2.4.1), and after 1 h, 0.6% of acetic acid solution (0.1 mL/10 g) was injected intraperitoneally. The number of writhings was counted every 5 min during 20 min of experiment.

##### Antinociception Evaluation: Tail-Flick Assay

The aim of this assay is to evaluate the nociception by a spinal cord stimulation activated by a tail thermoperception [45,46]. Initially, the animals were maintained in acrylic restraints, whereas a part of the tail (5 cm from the basis) was exposed to the heat from a projector lamp (55 ± 1 °C) in an Analgesimeter (Onda Científica, Campinas, Brazil). To avoid thermal injuries, the exposure to the tail heat was established at a maximum of 20 s (cut off). The analgesic activity was assessed by the area under the curve (AUC) or the analgesic effect, according to [47].

### 2.5. Statistical Analysis

Results were expressed as mean ± SEM from at least 5–6 animals per group and statistical analysis for peritonitis induced by carrageenan, formalin assay, and acetic-acid-induced writhing were performed using one-way analysis of variance (ANOVA) followed by the Tukey–Kramer posterior test. The tail flick assay was examined by two-way analysis of variance (ANOVA) followed by the Bonferroni posteriortest. Statistical significance was set at *p* < 0.05.

## 3. Results and Discussion

### 3.1. Drug Delivery System’s Performance: In Vitro Assays

#### 3.1.1. Kinetics Experiments

**Figure 2:**
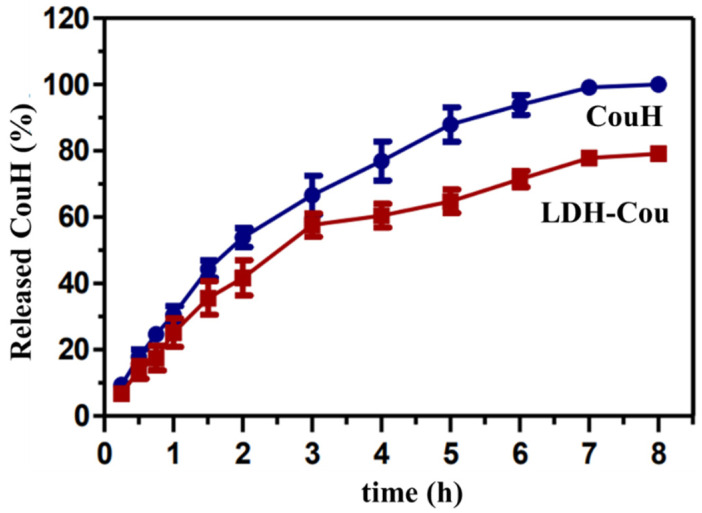
Release profile of CouH and LDH-Cou. Data are presented as percentage of released compound through time, mean ± SD (*n* = 5).

The release profile of non-intercalated (CouH) and intercalated coumarate (LDH-Cou) was determined by the amount of coumarate released from the donor compartment during 8 h (Figure 2). The acceptor solution, containing 30% propylene glycol in phosphate buffer (pH = 7.5) was used to mimic the intestinal fluids, where this class of compounds is preferentially absorbed by the organism.

Figure 2 displays the percentage of non-intercalated (CouH) and intercalated (LDH-Cou) coumarate released through time. There was a decrease in coumaric acid release when confined between the layers of the LDH in contrast of the non-intercalated phytochemical in the first 4 h of experiment. These data are confirmed by the statistic differences (*p* < 0.01) observed in the area under the curve (AUC 0–8 h) values for CouH (560.3 ± 54.6) and LDH-Cou (438.9 ± 47.4).

**Figure 3:**
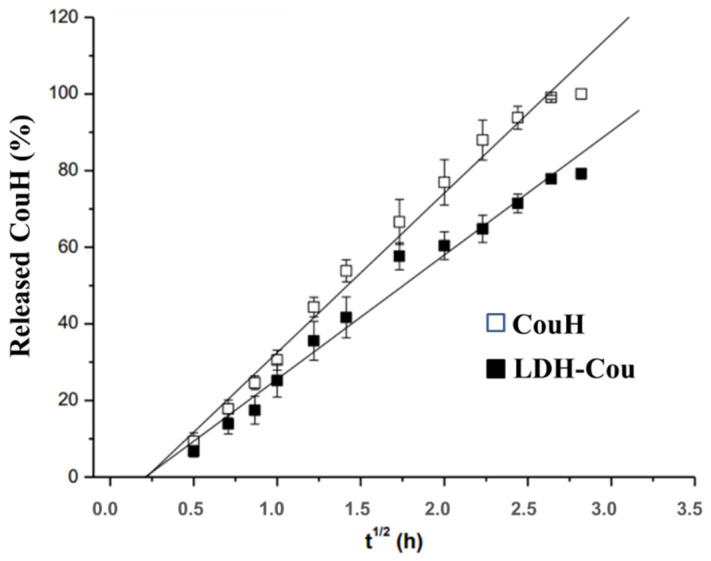
Higuchi model applied to the release kinetic curves of CouH and the intercalated species (LDH-Cou).

The curves of the release profile of both samples were evaluated by the Higuchi model (Figure 3). The CouH release ratio was the linear function of the square root of time, and the species is the only component that diffuses in the buffer media (Fick diffusion law) [38]. The flow values or the release constant (*k*) were 41.6 ± 1.5% h^−1/2^ (R^2^ = 0.99347) and 32.4 ± 1.5% ^−1/2^ (R^2^ = 0.98996) for CouH and LDH-Cou, respectively, indicating that the release of the intercalated species was slower, and followed the Higuchi diffusion model, due to the highest values of the determined correlation coefficient. Moreover, these results were statistically different (*p* < 0.01), showing that the prepared DDS formulation could modify (sustain) the coumarate release profile, in the locale of absorption.

Indeed, one of the main reasons to intercalate a drug in LDH is the expected modified release of that species from the inorganic carrier. The results observed in this work show similar profiles reported for other drugs such as naproxen [48], ibuprofen [49], diclofenac [50], and sulindac [51] intercalated into layered double hydroxides.

#### 3.1.2. Cytotoxicity Evaluation by the Neutral Red Assay

**Figure 4:**
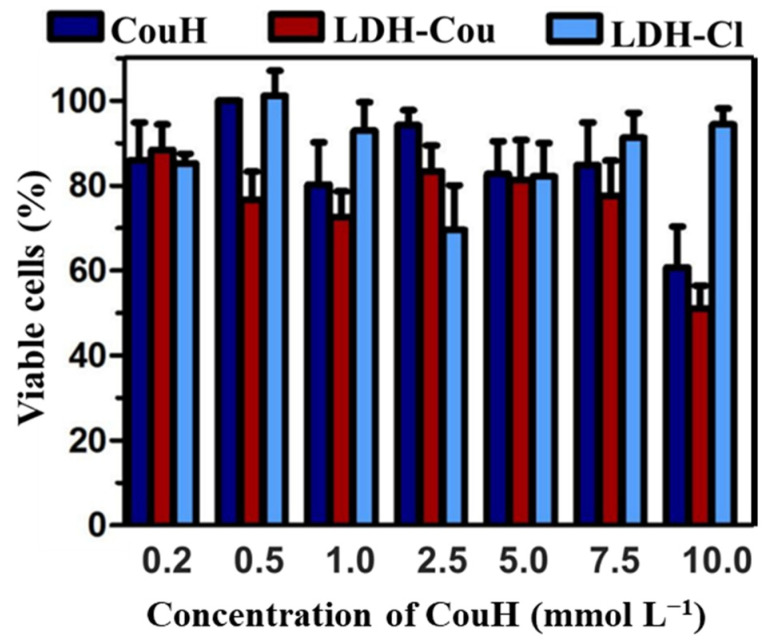
Viable cell percentage. Results extracted post-treatment with different concentration of CouH, LDH-Cou, and LDH-Cl (as reported in Section 2.3.2), evaluated by neutral red incorporation, average ± SD (*n* = 6).

The cytotoxicity tests evaluate the toxic effects of a substance over cultured cells. In this work, NIH 3T3 fibroblasts were used, a well-accepted cell line in the literature for its easy and reproducible results. NR was used to distinguish viable and dead cells. Figure 4 shows the percentage of the viable cells, i.e., those incorporating NR, in the concentration range of 0.2 to 10.0 mmol L^−1^ of coumaric acid. No higher concentrations could be tested, because LDH particles were not dispersed or suspended in the culture (DMEM) media.

The treatment with LDH-Cou and CouH were similar in terms of cell viability percentages, which means that formulations, especially from 0.2 to 7.5 mmol L^−1^ (with cell viability percentages above 70%), are not cytotoxic (Figure 4). At 10 mmol L^−1^, the treatment of cells with LDH-Cou and CouH evoked more pronounced cytotoxic effects (about 50% of cell viability), which was not observed for LDH-Cl, used as the control group. However, no significant differences were observed between LDH-Cou and CouH, indicating the low cytotoxic effects of CouH. This positive result was similar to those of the cytotoxicity (MTT and haemolysis) tests performed for mefenamic acid intercalated into LDH [32], revealing the low cytotoxicity of the LDH carrier. Considering the maximum dose used in vivo assay (30 mg/kg), the CouH dose administrated in each animal (approximately 30 g) was 0.9 mg or 5.5 mmol L^−1^, which is coherent with the concentration range in the viability cell assay, establishing a possible relationship between both assays.

### 3.2. In Vivo Pharmacological Assays

The in vivo pharmacological assays evaluated the performance of CouH and LDH-Cou compared with Ind, a commonly used NSAID for these assays, aside from the assessment of the participation of other mechanisms involved in the pharmacological response, especially the opioid system (controlled by Morph and Nalox) and the signalling of cAMP with Caffe.

#### 3.2.1. Anti-Inflammatory Evaluation: Peritonitis Induced by Carrageenan

**Figure 5:**
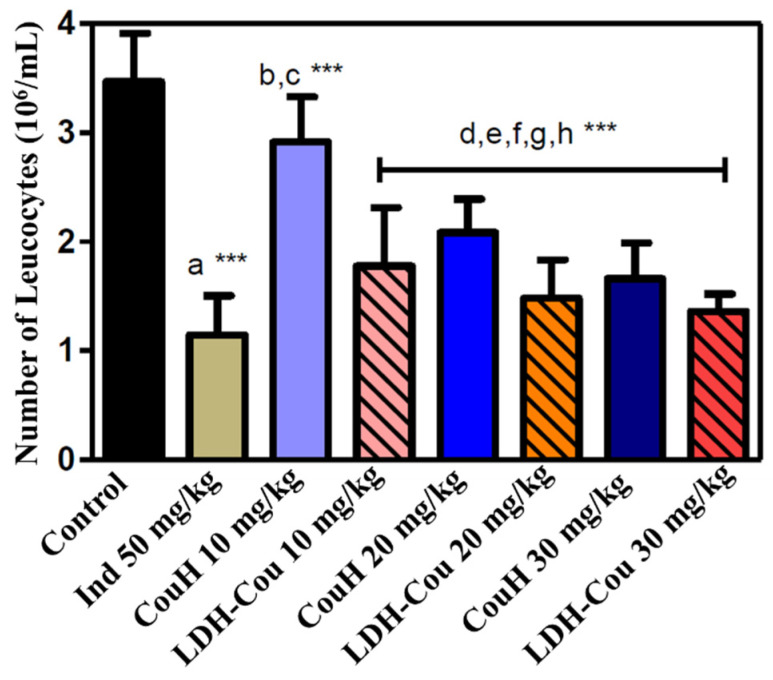
Number of leukocytes/mL at the pleural liquid post-treatment with CouH or LDH-Cou at 10, 20, and 30 mg/kg, compared to indomethacin (Ind, 50 mg/kg). Statistics: a—Ind 50 mg/kg vs. control; b—CouH 10 mg/kg vs. Ind 50 mg/kg; c—Cou-H 10 mg/kg vs. LDH-Cou 10 mg/kg; d—LDH-Cou 10 mg/kg vs. control; e—CouH 20 mg/kg vs. control; f—LDH-Cou 20 mg/kg vs. control; g—Cou-H 30 mg/kg vs. control; h—LDH-Cou 30 mg/kg vs. control.*** *p* < 0.001 (average ± SD, *n* = 7/group).

The peritonitis assay allowed to study the inflammatory process (leukocytes) induced by carrageenan injection (a pain agent) in the pleural exudate [47]. Figure 5 shows the results obtained for the post-treatment of Ind, CouH, and LDH-Cou at different doses. The treatment with LDH-Cou significantly reduced the number of leukocytes compared to CouH in the applied dose of 10 mg/kg (*p* < 0.001). However, in this dose, neither CouH nor LDH-Cou showed effects comparable to that of Ind, which evoked 1.5 times higher anti-inflammatory effect than CouH. CouH and LDH-Cou samples only reached similar effects to that of the clinical Ind dose at the higher concentration tested (30 mg/kg).

##### Anti-Inflammatory and Analgesic Evaluation: Formalin Assay

**Figure 6:**
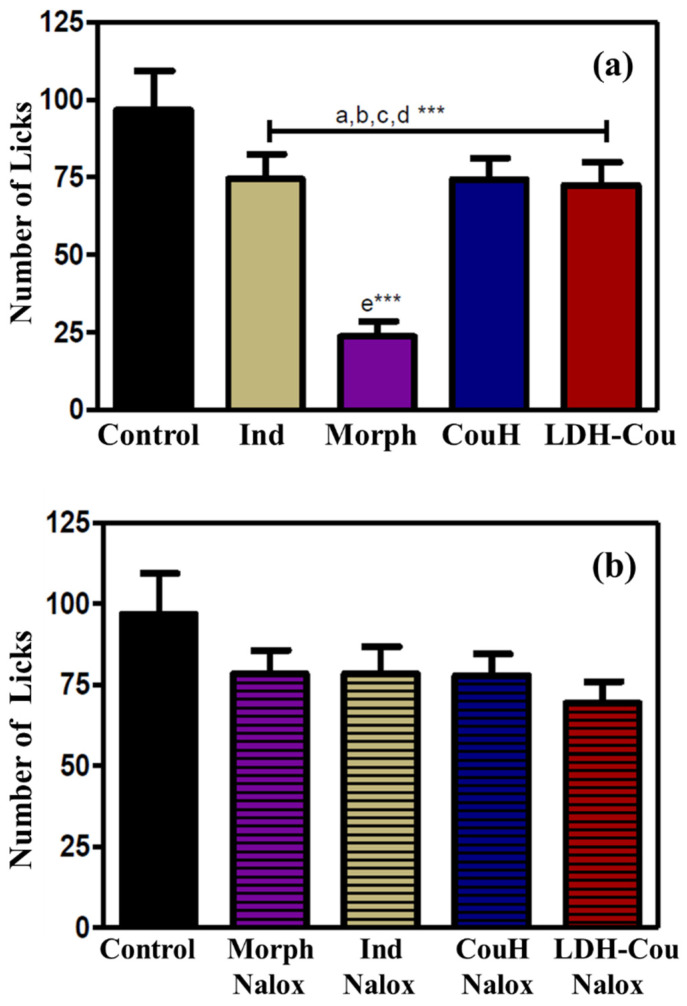

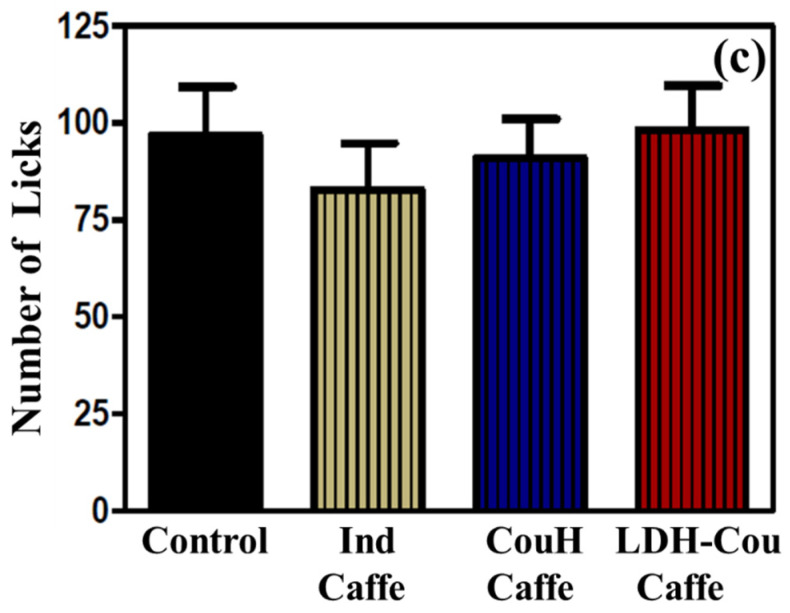
Formalin assay response from 0 to 5 min. (**a**) Groups without drug association; (**b**) Naloxone (Nalox) drug association; (**c**) caffeine (Caffe) drug association. Statistical analysis with significance set at *** *p* < 0.001 (ANOVA), where a—Ind vs. control; b—Morph vs. control; c—CouH vs. control; d—LDH-Cou vs. control; and e—Morph vs. Ind.

The formalin assay was used to evaluate the response of the formulations to a chemical inflammatory stimulus that indicated neurogenic pain and inflammation [5,9,10]. The data were expressed by the number of pain manifestations that were counted from 0 to 5 min and from 15 to 30 min. To investigate the mechanism involved in the possible pharmacological effects of CouH, formalin was applied by intraplanar injection, to directly activate nociceptive neurons. The initial response—from 0 to 5 min—is neurogenic, and it can be only blocked by Morph, resulting in a decrease of pain behaviour (lick time), as can be seen in Figure 6a [52]. After administration of Nalox, an antagonist of opioid receptors, the Morph action was blocked, reversing the analgesic activity (Figure 6b). The administration of 10 mg/kg of Caffe was used to verify the participation of the adenosine system in the response, as shown in Figure 6c.

The initial step of the assay (0–5 min) showed that the administration of Morph, Ind, CouH, and LDH-Cou reduced the behaviour representative of neurogenic pain, whereas only the Morph effect was reversed by the Nalox administration. These results suggest that the initial step of the test did not have the participation of either the opioid mechanism or adenosine in the induction of the pharmacological effects of CouH.

**Figure 7:**
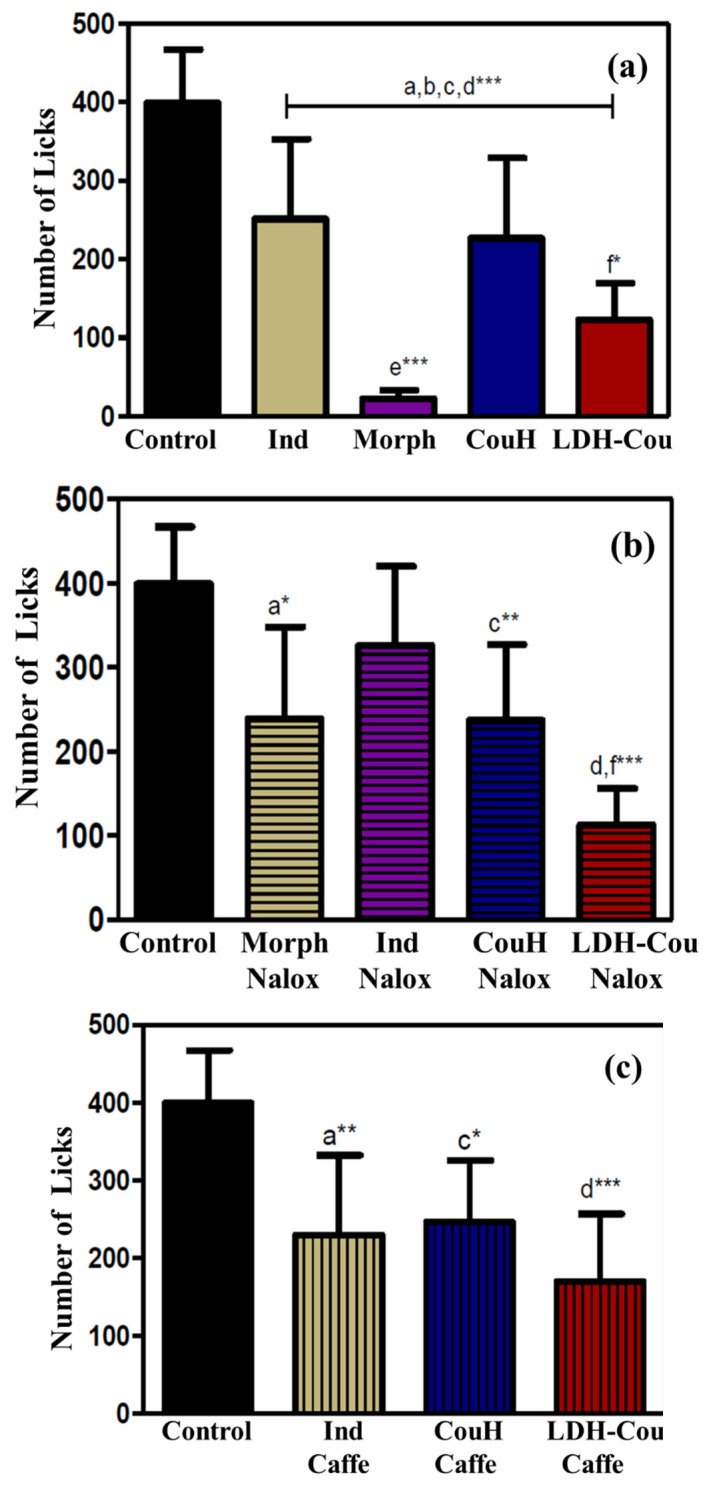
Formalin assay response, from 15 to 30 min. (**a**) Groups without association; (**b**) groups associated with naloxone (Nalox); (**c**) groups associated with caffeine (Caffe). Statistical analysis with significance set at *** *p* < 0.001; ** *p* < 0.01; and * *p* < 0.05 (ANOVA), where a—Ind vs. control; b—Morph vs. control; c—CouH vs. control; d—LDH-Cou vs. control, e—Morph vs. Ind; and f—CouH vs. LDH-Cou.

Figure 7 shows the second step of the test, in the time range of 15–30 min and reflecting the inflammatory pain [52]. All groups showed anti-inflammatory effects with statistical difference compared to the control group (*p* < 0.001), as shown in Figure 7a. At this point, CouH demonstrated similar response to Ind, while LDH-Cou induced more pronounced effects (*p* < 0.05), indicating that intercalation of coumarate into LDH can enhance its local anti-inflammatory effect.

Regarding the third phase test, from 15 to 30 min, neither Nalox nor Caffe administrations were able to revert the pharmacological effects of Ind or CouH (Figure 7a,b). Moreover, the participation of neither the opioid nor the adenosine pathway was observed in the anti-inflammatory mechanism. Therefore, it can be concluded that the anti-inflammatory mechanism of CouH is like that of NSAIDs, possibly the inhibition of cyclooxygenase [23,53], and that the intercalation of coumarate into LDH did not change these mechanisms.

##### Anti-Inflammatory and Analgesic Evaluation: Acetic-Acid-Induced Writhing and Tail-Flick Assay

**Figure 8:**
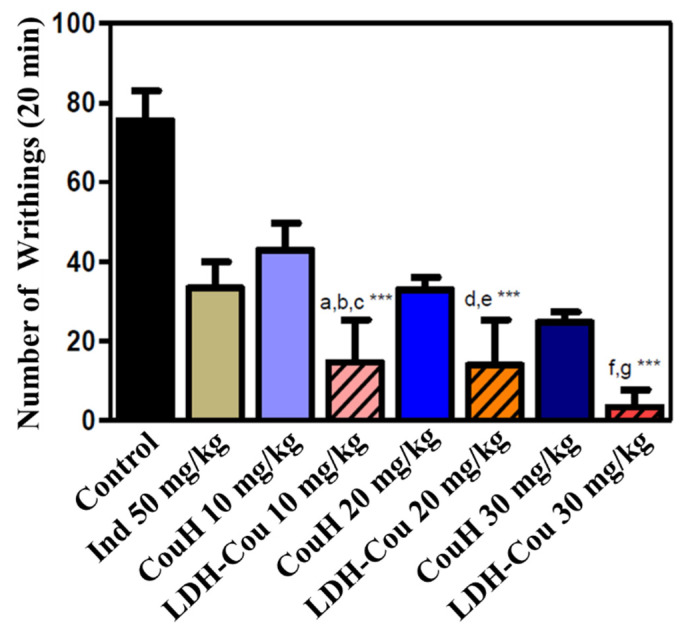
Acetic-acid test. Number of writhings in 20 min of experiment. a—LDH-Cou 10 mg/kg vs. Ind 50 mg/kg; b—LDH-Cou 10 mg/kg vs. CouH 10 mg/kg; c—LDH-Cou 10 mg/kg vs. CouH 20 mg/kg; d—LDH-Cou 20 mg/kg vs. Ind 50mg/kg; e—LDH-Cou 20 mg/kg vs. CouH 20 mg/kg; f—LDH-Cou 30 mg/kg vs. Ind 50mg/kg; g—LDH-Cou 30 mg/kg vs. CouH 30 mg/kg. *** *p* < 0.0001 (average ± SD, *n* = 7/group).

The induced writhing assay was performed to evaluate CouH and LDH-Cou’s effects over an acute chemical painful stimulus, such as the intraperitoneal injection of acetic acid. After the application of the harmful agent, the number of writhings in relation to the control (saline and Ind solution) was counted, and the treatments were applied. Figure 8 shows the number of writhings obtained for each group tested during 20 min of observation.

The results showed that the antinociceptive effect of CouH, at the three doses applied, was similar to that obtained with Ind treatment. As for the LDH-Cou, it demonstrated a significant reduction (*p* < 0.001) in the number of writhings when compared to Ind, specially at the highest dose (30 mg/kg). Moreover, the intercalated CouH, at 10 mg/kg, induced a higher analgesic effect (*p* < 0.001) than that evoked by CouH at 20 mg/kg. At 20 mg/kg, LDH-Cou evoked a similar effect to CouH at 30 mg/kg. The results obtained in this work pointed out that the intercalation in LDH enhanced the antinociceptive effects of coumarate, probably due to mechanisms like those of the NSAIDs, such as Ind in association with the drug release modulation by inorganic carrier.

Just a few works showed in vivo tests to evaluate the anti-inflammatory effects of such formulations [31,32,33]. Bonina et al. [50] performed an assay with a gel containing LDH with diclofenac for topic application in humans, to decrease cutaneous erythema, and found higher efficiency of the intercalated compound when compared to free diclofenac. Moreover, assays with LDH containing ibuprofen and ketoprofen were also performed to demonstrate the reduction of the side effects in the gastrointestinal tract, frequently observed after a prolonged treatment with non-intercalated drugs [54,55].

Regarding CouH’s pharmacological effects, some studies have discussed its potential anti-inflammatory and antioxidant properties [21,56,57,58] as well as antifungal activity [59]. However, the LDH carrier containing coumarate was only reported in a few studies [60,61], which were devoted to their preparation and characterization for analytical purposes, by using different LDH composition. All these observations highlight the pharmacological potential of the results presented in this study.

**Figure 9:**
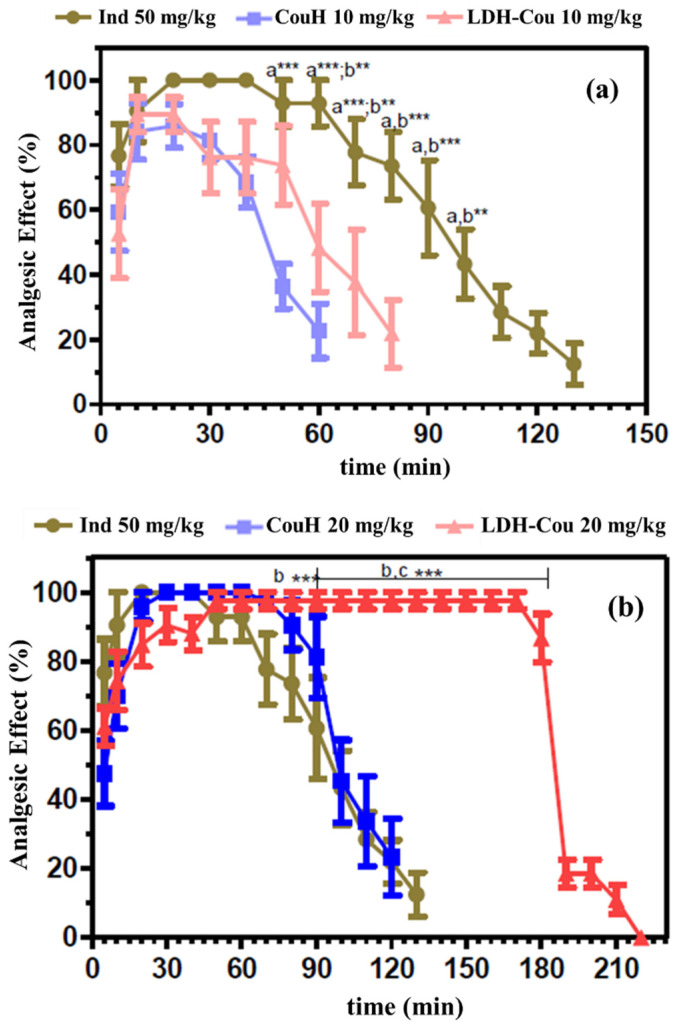

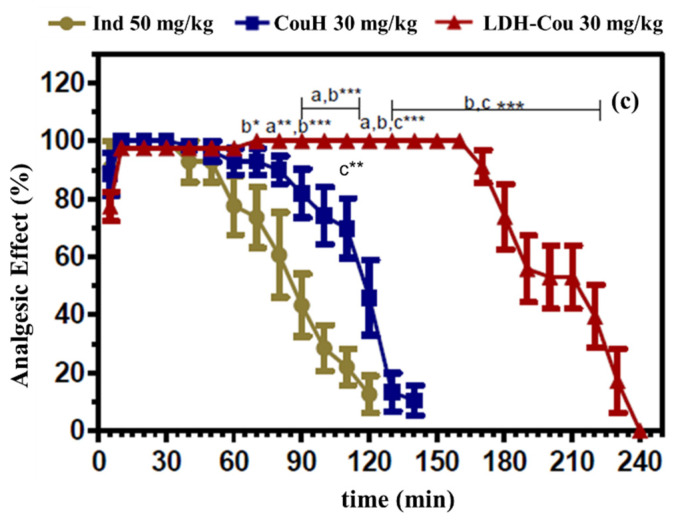
Profiles with maximum percentage of the analgesic effect versus time obtained after treatment with CouH or LDH-Cou, in the doses 10 mg/kg (**a**), 20 mg/kg (**b**) and 30 mg/kg (**c**), trough the tail-flick test. Statistics: a—Ind vs. CouH; b—Ind vs. LDH-Cou; c—CouH vs. LDH-Cou; * *p* < 0.05; ** *p* < 0.001; and *** *p* < 0.0001, two-way ANOVA (average ± SEM, *n* = 7/group).

The tail-flick assay revealed the analgesic response of the animals to the tested compounds and formulation (Figure 9); the results are represented in plots separated according to the dose and their comparison to the control, Ind at 50 mg/kg.

The analgesic activity of CouH, LDH-Cou, and Ind was summarized in Table 1 by the values of the area under the curve (AUC_0-240_) and time of anaesthesia recovery (T_rec_).

The results in Table 1 and Figure 9 pointed out that either CouH or LDH-Cou in the doses 20 and 30 mg/kg induced a significantly higher (*p* < 0.001) intensity and duration of the analgesic effect than Ind. Moreover, LDH-Cou increased the intensity and prolonged the time of analgesia by 1.7 times compared to CouH and 1.8 times in relation to Ind. Although the investigation of the molecular pathways responsible for the response of the bioactive organic is out of the scope of this work, several studies reported the advantages of drug intercalation into LDH: improved stability and superior water solubility of bioactive species in biological environments, lower-dose administration, and modified drug release [26]. The intercalation of CouH could improve its limited physicochemical properties such as chemical stability and low aqueous solubility. For instance, anticancer drugs intercalated into LDH showed better performance than the non-intercalated species, suggesting a protective effect of the carrier [62]. A similar proposition can justify the enhanced antinociceptive activity of intercalated coumarate: its chemical stability (related to the antioxidant property) may be prolonged because it is protected from reactions in the organism that can decompose it during the transport, in comparison to the non-intercalated CouH.

As observed for some bioactives [63], another advantage of intercalation could be to increase the aqueous solubility of CouH, which was achieved considering the more pronounced pharmacological effects obtained after animal treatment with LDH-Cou, suggesting an increase in coumarate bioavailability. The zeta potential of nanoparticles can also be considered in the discussion about the superior biological results of the hybrid material compared to the non-intercalated CouH. Particles can quickly hold to the cell’s surface because of the efficacy of interactions among them [64]. The LDH-Cou particles have positive zeta potential (see Supplementary Material), which can promote a strong interaction with the negatively charged cell membrane if compared to CouH, present as an anion (its deprotonated form) in the physiological pH values. The chemical composition of the layers is an additional factor that can add functionality to the LDH hybrids [35,36]. Zinc ions present valuable properties for the organism such as antioxidant, anti-inflammatory, and antinociceptive activities [65,66,67], which can also be considered in the analysis of the experimental results.

## 4. Conclusions

The pharmacological evaluation demonstrated that the mechanism by which CouH exerts its anti-inflammatory and analgesic activities is similar to those of Ind and NSAIDs of commercial application. Furthermore, the intercalation of coumarate into LDH potentialized its effects and increased the analgesic duration in relation to the non-intercalated (CouH) or Ind. This pointed out a novel DDS with low toxicity and greater pharmacological efficacy for therapeutic purposes.

## Figures and Tables

**Table 1:** Antinociceptive tests. Area under the curve and analgesic time determined in the tail-flick essay. Statistics: a—CouH 10 mg/kg vs. Ind; b—LDH-Cou 10 mg/kg vs. Ind; c—CouH 10 mg/kg vs. LDH-Cou 10 mg/kg; d—LDH-Cou 20 mg/kg vs. Ind; e—CouH 20 mg/kg vs. LDH-Cou 20 mg/kg; f—CouH 30 mg/kg vs. Ind; g—LDH-Cou 30 mg/kg vs. Ind; h—CouH 30 mg/kg vs. LDH-Cou 30 mg/kg, where ** *p* < 0.01; and *** *p* < 0.001 (average minimum and maximum, *n* = 7/group).

Samples	AUC_0-240_	T_rec_ (min)
Ind—50 mg/kg	106.0 (92.0–114.5) ^d,f,g^ ***	120 (90–130) ^d,g^ ***
CouH—10 mg/kg	53.5 (33.5–54.5) ^a^ ***	50 (40–60) ^a^ ***
LDH-Cou—10 mg/kg	74.5 (52.5–84.0) ^b^ ***^; c^ **	70 (60–80) ^b,c^ ***
CouH—20 mg/kg	111.0 (97.0–117.0) ^e^ ***	110 (100–120) ^e^ ***
LDH-Cou—20 mg/kg	194.0 (190.0–201.0)	190 (190–210)
CouH—30 mg/kg	142.5 (110.0–146.0) ^h^ ***	130 (120–140) ^h^ ***
LDH-Cou—30 mg/kg	220.0 (210.5–242.0)	220 (210–230)

## Data Availability

Raw data were generated at University of São Paulo (USP) and State University of Campinas (UNICAMP). Derived data supporting the findings of this study are available from the corresponding author V.R.L.C., on request.

## Supplementary Materials

The following supporting information can be downloaded at https://www.mdpi.com/article/10.3390/pharmaceutics14050934/s1, Figure S1: UV–VIS spectra of *p*-coumaric acid at 288 nm, Figure S2: Calibration curve of *p*-coumaric acid obtained from UV–VIS spectra in different concentrations (*n* = 6), Figure S3: Particle size distribution of LDH-Cou sample.
