# Supplementary material for: Anti-Inflammatory and Analgesic Evaluation of a Phytochemical Intercalated into Layered Double Hydroxide
Source: Pharmaceutics. 2022 Apr 25;14(5):934. doi: 10.3390/pharmaceutics14050934 (PMC9144641; doi:10.3390/pharmaceutics14050934)
Supplement: Supplementary file 1 [file pharmaceutics-14-00934-s001.zip › pharmaceutics-1657375-supplementary.pdf]

Supplementary Material

Anti-inflammatory and Analgesic evaluation of a phytochemical intercalated into Layered Double Hydroxide

Viviane A. Guilherme[1,2], Vanessa R. R. Cunha[3,4], Eneida de Paula[1], Daniele R. de Araujo[5*], Vera R. L. Constantino[3*]

[1] Departamento de Bioquímica e Biologia Tecidual, Instituto de Biologia, Universidade Estadual de Campinas, UNICAMP, 13083-862, Campinas, São Paulo, Brazil. viguilherme@gmail.com (V.A.G.), depaula@unicamp.br (E.P.)

[2] Faculdade de Farmácia, Universidade Adventista de São Paulo, UNASP, 13448-900, Engenheiro Coelho, São Paulo, Brazil.

[3] Departamento de Química Fundamental, Instituto de Química, Universidade de São Paulo, USP, Av. Prof. Lineu Prestes 748, 05508-000, São Paulo, São Paulo, Brazil.

[4] Instituto Federal de Educação, Ciência e Tecnologia de Mato Grosso - Campus Juína, IFT-MT, Juína-MT, Brasil. vanesssa.roberta@ifmt.edu.br (V.R.R.C)

[5] Centro de Ciências Naturais e Humanas, Universidade Federal do ABC, UFABC, 09210-170, Santo André, São Paulo, Brazil.

* Correspondence: daniele.araujo@ufabc.edu.br (D.R.A.); vrlconst@iq.usp.br (V.R.L.C.)

1. Materials and Methods

1.1. Reagents

p-coumaric acid ($C_9H_8O_3$, Sigma), aluminum chloride hexahydrate ($AlCl_3 \cdot 6H_2O$, Aldrich), zinc chloride ($ZnCl_2$, Aldrich), sodium hydroxide (NaOH, Merck), caffeine (Sigma-Aldrich), naloxone (Sigma-Aldrich), penicillin (Nutricell), neutral red (Sigma-Aldrich), phosphate buffer saline (PBS), Turk´s solution, and Dulbecco´s Modified Eagle Medium (DMEM, Nutricell) were used without further purification.

1.2. Spectrophotometric determination of p-coumaric acid concentration

The wavelength of maximum coumaric acid absorption in sodium chloride aqueous solution (NaCl 0.9 %), the UV-VIS spectra of CouH solution concentration from 10 to 50 µmol L^{-1} were recorded in the 200 to 500 nm range, using a cuvette of 1 cm of optical pathway (Figure S1). The spectra were recorded in a spectrophotometer UV-VIS (Varian Cary 50 / Bio-Tek ELx 800) and values were analyzed by Graph Pad Software, Inc., USA and the Beer´s Law was applied.

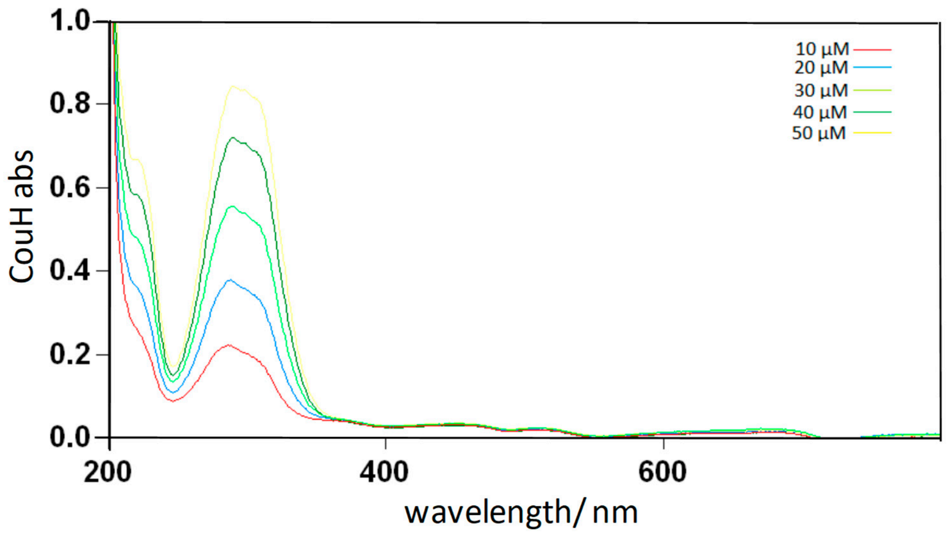

Figure S1. UV-VIS spectra of coumaric acid in different concentrations in sodium chloride aqueous solution (0.9 % NaCl).

The calibration curve obtained at 288 nm, Figure S2, provided the molar absorptivity of coumaric acid, 1907 L mol^{-1} cm^{-1} with correlation coefficient (R^2) of 0.99969 (y = -0.00404 + 0.01907) and it was used to determine the concentration of coumarate delivery in time, as well, to calculate the delivery constant.

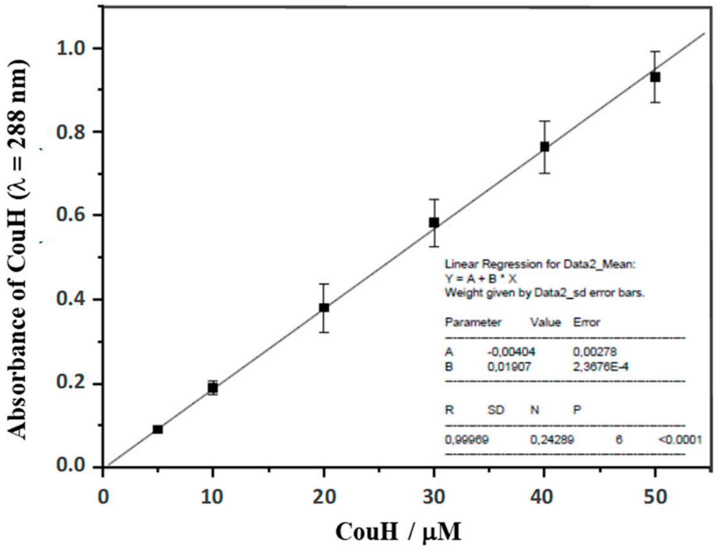

Figure S2. Calibration curve of coumaric acid obtained by UV-VIS spectrophotometry at different concentrations (n= 6).

Zeta potential and particle size were measured with Zetasizer NanoZS, Malvern Instruments, Worcestershire, UK. The mean hydrodynamic diameter of the suspension of LDH-Cou obtained by Dynamic Light Scattering (DLS) is 226 nm (PdI: 0.419). It can be noticed the slight particle size variation (Figure S3), obtaining a mono-modal system with a similar particle size population. Moreover, LDH-Cou shows low tendency towards agglomeration since its zeta potential is + 39 mV, which indicates a good stability of the aqueous dispersion of the material.

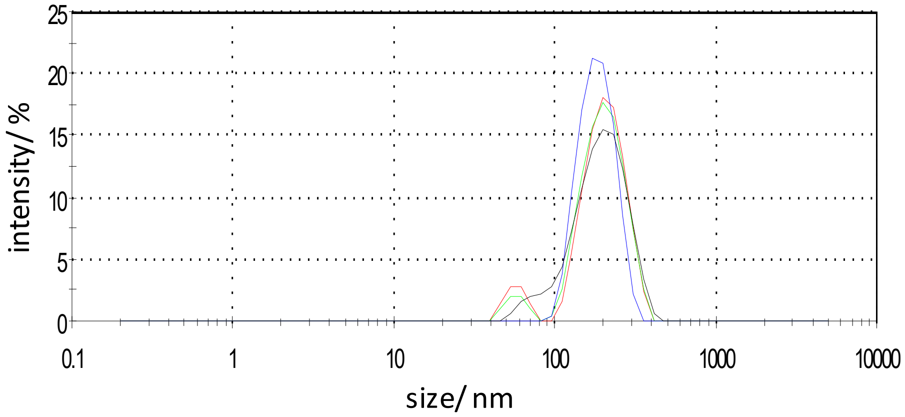

Figure S3. Particle size distribution of LDH-Cou sample.
